# Building bridges between doctors and patients: the design and pilot evaluation of a training session in argumentation for chronic pain experts

**DOI:** 10.1186/s12909-015-0374-6

**Published:** 2015-05-19

**Authors:** Claudia Zanini, Piercarlo Sarzi-Puttini, Fabiola Atzeni, Manuela Di Franco, Sara Rubinelli

**Affiliations:** 1 Department of Health Sciences and Health Policy, University of Lucerne and Swiss Paraplegic Research, Lucerne, Nottwil Switzerland; 2 Swiss Paraplegic Research, Lucerne, Nottwil Switzerland; 3 Rheumatology Unit, L. Sacco University Hospital, Milan, Italy; 4 Department of Internal Medicine, Sapienza University of Rome, Rome, Italy

## Abstract

**Background:**

Shared decision–making requires doctors to be competent in exchanging views with patients to identify the appropriate course of action. In this paper we focus on the potential of a course in argumentation as a promising way to empower doctors in presenting their viewpoints and addressing those of patients. Argumentation is the communication process in which the speaker, through the use of reasons, aims to convince the interlocutor of the acceptability of a viewpoint. The value of argumentation skills for doctors has been addressed in the literature. Yet, there is no research on what a course on argumentation might look like. In this paper, we present the content and format of a training session in argumentation for doctors and discuss some insights gained from a pilot study that examined doctors’ perceived strengths and limitations vis-à-vis this training.

**Methods:**

The training session (eight hours) combined different aspects from prominent theories of argumentation and was designed to strengthen doctors’ argumentative discussion skills. A convenient, self-selected sample of 17 doctors who were experts in the field of chronic pain participated in the training and evaluated it via a feedback form and semi-structured interviews.

**Results:**

The participants found that the training session gave a structure to types of communication they use to interact with their patients, and taught them techniques that can increase their effectiveness. Moreover, it provided tools to help address some of the challenges of modern doctor–patient interactions, including dealing with patients’ unrealistic expectations and medically inaccurate beliefs, and reaching agreement when there are differences of opinion.

**Conclusions:**

This study enriches the research in the field of medical education. In line with the findings of studies that explore the value of argumentation in different fields, argumentative discussion skills can be applied by doctors to express their views and to account for the views of patients without patronizing the interaction. In this paper, we provide a basis to reflect on the value of argumentation in enhancing patients’ right to autonomy and self-determination in interactions with their doctors.

## Background

The value of communication in doctor–patient interactions is well established, and communication is thus recognized as an integral part of clinical practice [1, 2]. In recent years, communication has been considered not only a matter of professional experience or personal inclination but also a set of behaviors that can be taught and learned [3]. As a consequence, efforts in implementing communication courses in medical curricula have increased [4, 5].

One way to define the focus of medical training in communication is to reflect on what the current research on doctor–patient communication has underlined as the main challenges. The literature has acknowledged a change in the preferred models of doctor–patient communication, from a paternalistic to a shared decision–making model [6, 7], where both parties participate by sharing information about treatment options and negotiate to reach a final agreement [8]. However, as we show elsewhere [9, 10], a shared decision–making model requires doctors to be able to support their viewpoint with reasons that are understandable and relevant to patients, to identify and address suboptimal viewpoints (e.g., patients’ medically inaccurate beliefs), and to negotiate when patients have a different but sustainable viewpoint.

In light of this, we infer that doctors would benefit from a training course in argumentation. As defined by van Eemeren and Grootendorst [11], argumentation is “a verbal, social, and rational activity aimed at convincing a reasonable critic of the acceptability of a standpoint by putting forward a constellation of propositions justifying or refuting the proposition expressed in the standpoint.” Argumentation is a communication process where the speaker, through the use of reasons, aims to convince the interlocutor of the acceptability of a viewpoint.

The value of argumentation as an important communication process is well established in many fields, including education (e.g., [12, 13]), law (e.g., [14, 15]), politics (e.g., [16, 17]), and artificial intelligence (e.g., [18, 19]). A number of argumentation and health communication scholars have recently supported the potential of argumentation theory as a topic for the training of health professionals in terms of how argumentation is used and can be used to enhance doctor–patient communication (e.g., [10, 20–26]). In particular, argumentation is fostered because it can help clarify patient expectations and enhance patient participation and shared decision–making in light of its supposed impact on consultation outcomes, such as patient adherence and satisfaction [26]. Despite the existence of studies showing the value of argumentation in doctor–patient interactions, there is no evidence that a training course for health professionals exists, and there is no description in the literature of what such a course might look like.

Argumentative discussion skills seem to be of particular value in those fields of medicine in which patients have strong viewpoints on their health conditions and on what can be done. Indeed, as mentioned above, argumentation is a particularly important communication process when interlocutors hold different viewpoints and there is a need to reach an agreement. Chronic pain is one of the areas in which doctors and patients might have a difference of opinion [27]; due to patients’ prolonged experience in dealing with pain, they have views to share with their health professionals that are often shaped by their experiences, norms, and values, and by information from various sources, such as the Internet [28, 29]. Patients’ health literacy and empowerment are acknowledged to enhance the sharing of information and decisions between doctors and patients and to improve the self-management of disease and treatment [30–32]. However, dealing with empowered and health literate patients is not always an easy task for health professionals, as communication can be characterized by strong disagreement, which can lead to frustration, disappointment, and even anger [33, 34].

As part of a project on argumentation in doctor–patient interactions, we developed a training course in argumentation targeting doctors who are active in the field of chronic pain. The objective of this paper is to present the design and pilot evaluation of this training course. More specifically, we present the content and format of the course in detail, and to further reinforce the claim that such a course could be a promising part of medical education, we discuss some insights gained from a pilot study aimed at identifying doctors’ perceived strengths and limitations of this training in argumentation.

## Methods

### Design principles of the course in argumentation for doctors

#### Pedagogical foundations

The training course was designed to strengthen doctors’ skills in argumentation. Given that argumentation is a natural communication process that human beings practice in different contexts [35], the main purpose of the course was to increase doctors’ awareness of when and how they argue and to provide them with instruments to make better choices when engaging in argumentation with patients. To achieve this, the course had two main goals that are in line with current advice on how to teach argumentation (see, specifically, [36, 37]). First, at the cognitive level, it aimed to foster an understanding of what argumentation is, why it matters in doctor–patient communication, how argumentation can be constructed, and what the facilitators and barriers are to reaching agreement when there is a difference of opinion. Second, at the behavioral level, it aimed to exemplify how this theoretical understanding can be transferred to the praxis level, namely doctor–patient communication, to deal with issues where agreement can be reached through the exchange of reasons.

#### Theoretical frameworks

The content of this training course was not developed following one specific approach to argumentation. In light of the pedagogical foundations of the course, its content was developed in an eclectic way, by bringing together different aspects of the most prominent theories of argumentation. There were two main criteria for selecting these different aspects: first, their applicability to doctor–patient communication and second, the fact that their understanding would not require prior knowledge of specific fields of communication sciences (e.g., linguistics or pragmatics), which supposedly doctors do not have.

The content of the course was structured in the following five modules:

1. Argumentation in doctor–patient communication
2. The structure of argumentation
3. Argument schemes
4. Evaluation of argumentation and fallacies
5. Argumentation in the context of persuasion

Below is an illustration of the specific content and theoretical frameworks of each module. This content will be exemplified by referring back to the following case of doctor–patient argumentation based on the study by Rubinelli [38]:

Doctor: *Take this antibiotic. You will see that in less than a week you will feel better.*

Patient: *Mmm … I am not keen on taking antibiotics. I just saw a program about antibiotic resistance.*

Doctor: *Right, antibiotic resistance can be a problem when there is an overuse of antibiotics. But, this is not your case. When was the last time you took an antibiotic?*

Patient: *Actually, years ago.*

Doctor: *See? You can go ahead with antibiotics. No worries.*

1. Argumentation in doctor–patient communication

Argumentation was introduced to the doctors in this study using the definition given by van Eemeren and Grootendorst (see the introduction of this paper [11]). In the above example, the doctor acknowledges that the patient has concerns about the use of antibiotics and supports his standpoint (“Take this antibiotic”) with reasons related to the fact that the patient should not worry about antibiotic resistance. Major emphasis was placed on distinguishing between “argumentation” (where the speaker has the intention to convince the interlocutor of the acceptability of a standpoint) and “explanation” (where the speaker aims to describe the features of a phenomenon to make sense of it) on the basis of the conceptualization given by Snoeck Henkemans [39]. Argumentation was then contextualized for its relevance in doctor–patient communication on the basis of recent studies that focus on the value of argumentation in enhancing shared decision–making [10, 20–22, 40, 41].

2) The structure of argumentation

The second module of the course was devoted to explaining the structure of argumentation to identify the type of differences of opinion at stake, the standpoints and the premises expressed by the interlocutors. In the example above, there is a mixed difference of opinion, where the doctor put forwards a standpoint (“Take antibiotics”) that the patient, in the first instance, rejects (“I am not keen on taking antibiotics”). The theory behind this module was borrowed from van Eemeren and colleagues [36].

3) Argument schemes

An argument scheme is a strategy of argumentation that reveals the link between the premises and a standpoint to be defended [42]. In the example above, the argumentation “Take antibiotics. In less than a week you will feel better” is based on a causal relationship between “taking antibiotics” and “feeling better”. Argument schemes were introduced to the doctors in the study using the framework developed by van Eemeren and colleagues [36] and by selecting specific argument schemes from Walton et al. [43] based on their occurrences in doctor–patient communication [25].

4) Evaluation of argumentation and fallacies

Module 4 was devoted to explaining the concept of the soundness of argumentation and that of “fallacies”. With reference to the example above, this module focused on how to evaluate whether the reasons given by the doctor support his standpoint regarding the need to take antibiotics and whether the reasons given by the patient support his unwillingness to take them within this context. Fallacies were introduced as suboptimal argumentative moves that can prevent or hinder the resolution of a difference of opinion under the framework given by van Eemeren et al. [36]

5) Argumentation in the context of persuasion

The last module addressed the nature of argumentation as rational persuasion, in line with the framework of Rubinelli [24, 38]. Participants were invited to reflect on the determinants of individuals’ (positive or negative) attitudes towards a standpoint, and on how to use argumentation to influence attitudes [44]. The concepts of central versus peripheral routes to persuasion, according to the Elaboration Likelihood Model [45], and their influencing factors were presented. With reference to the example above, the focus was on explaining the main role that aspects such as the relevance of the reasons put forward by the doctor (namely, the reasons not to worry about antibiotics resistance) have in the patient’s acceptance of the overall argument.

#### Course format

The course was structured in a five-hour morning session and a three-hour afternoon session. The morning session consisted of a frontal lecture with a 15-minute question and answer period after each module. In the afternoon session, doctors had to engage in a role-play based on scenarios developed by the teachers. Three scenarios were developed by reflecting on the findings of a parallel study focused on the difficulties doctors face in their communication with chronic pain patients [46]. More specifically, three written scenarios were presented to the doctors:

1. the case of a patient who risks making a wrong decision based on medically incorrect information;
2. the case of a patient who has a standpoint that differs from that of the doctor but that is based on valid reasons;
3. the case of a patient who disagrees with the doctor and is not willing to engage in any argumentative exchange.

During the afternoon session, the main emphasis was placed on the fact that doctors can use argumentation not only to inform patients’ decision–making and to correct patients’ medically inaccurate beliefs but also to reach mutual agreement in cases where argumentation ends with the doctor accepting the patient’s point of view even if it differs from his or her own. Indeed, in relation to the last point, autonomy in doctor–patient communication is also respecting patients’ right to self-determination provided that the conditions necessary for autonomous choice (e.g., adequate discussion of the pros and cons of the different viewpoints) are met [47]. Argumentation is a process that can foster the fulfillment of these conditions [48].

At the end of the course, the participants received a checklist, a list of questions doctors can use during medical consultation to support the integration of the argumentative principles taught in the course into daily practice (Table 1).

**Table 1:** Checklist provided to the participants at the end of the training course to support the integration of the argumentative principles taught in the course into daily practice

1.	In your opinion, what is your problem?
2.	In your opinion, what is the cause of your problem?
3.	Do you think that there is a connection between your problem and some aspects of your life? If yes, which one?
4.	Do you have any concerns in relation to your health condition?
5.	What is your biggest burden in relation to your health condition?
6.	What do you expect from a treatment?
7.	Have you heard about a treatment that you would like to try?
8.	Have you heard about a treatment that you would like to avoid?
9.	Have you already tried a treatment that met your expectations? If yes, which one?
10.	Have you already tried a treatment that fell short of expectations? If yes, which one?

### Selection of participants

The course took place during the “Giornate Reumatologiche Sannite” (Benevento, Italy), a major Italian event dedicated to education and exchange among Italian specialists in rheumatology. The course was organized as an eight-hour training session held by two of the authors of this paper (SR and CZ).

The study was approved by the Regional Ethics Committee of the Italian speaking part of Switzerland. All participants were volunteers and provided informed consent.

The recruitment of the participants was based on a convenient and self-selected sample of 17 doctors who were experts in the field of chronic pain, with the following characteristics (Table 2): six women and 11 men, mostly rheumatologists (n = 12) and working in a public hospital (n = 11), ranging in age from 34 to 73 years (mean = 54), with years of practice ranging from nine to 40 years (mean = 27). On average, they visited 49.4 patients per week (SD = 20.8).

**Table 2:** Participants’ characteristics

Characteristic	Number
Sex	
Male	11
Female	6
Age category	
30-40	1
41-50	4
51-60	9
61-70	2
71-80	1
Main specialty	
Rheumatology	12
Neurology	2
Psychiatry	1
Immunology	1
Nervous and mental diseases	1
Working place	
Public hospital	11
Private practice	3
Public hospital and private practice	3
Number of patients visited per week	
0-20	1
21-40	4
41-60	8
61-80	3
>80	1
Years of practice	
0-9	1
10-19	2
20-29	5
30-39	8
>39	1
Total	17

### Materials and procedure

Data on the aspects explained below were collected through paper-and-pencil questionnaires and interviews.

#### Socio-demographic data and communication with patients

Before the course, the participants completed a questionnaire to gather their socio-demographic characteristics and a questionnaire to self-assess their satisfaction, difficulties, and confidence in communicating with patients. The second questionnaire was composed of 1) a general item to evaluate the doctors’ satisfaction with communication with their patients, 2) an open-ended question on the difficulties encountered in communicating with their patients, and 3) 12 items to assess the doctors’ self-efficacy in communicating with their patients. The 12 items (Table 3) were an adaptation of the self-efficacy scale developed by Parle, Maguire, and Heaven [49] for specified communication tasks before and after training. The scale included the original items that highlight the doctors’ ability to initiate a discussion with patients about their concerns (question 1 in Table 3), to explore patients’ feelings (question 8), to break bad news (question 9), to help patients deal with the uncertainty of their situation (question 10), to assess symptoms of anxiety and depression (question 11), and to conclude a consultation with an agreed problem list and a plan for action (question 12). Our scale explored the item “manage collusion” by splitting it into three sub points to evaluate the nuances that collusion during a medical consultation can have (questions 5–7). Finally, our self-efficacy scale assessed two additional aspects, namely the doctors’ ability to capture patients’ perspective on their health conditions and treatments (questions 2–3) and to support medical recommendations with reasons (question 4). Participants were asked to rate “how confident you feel in your ability to successfully manage each of these situations” on a scale from 1 (totally confident) to 10 (not at all confident). Participants were also asked about communication courses they had taken in the past (e.g., which kind of course, when and for how long).

**Table 3:** Participants’ assessment of the communication with their patients before the training course

Item	Mean	SD
1. Initiate a discussion with a patient about his or her concern	2.75	±1.05
2. Elicit a patient’s viewpoint on health condition and treatments	2.94	±1.78
3. Elicit a patient’s reasons for this viewpoint	3.5	±1.33
4. Explain to a patient the reasons in support of a medical recommendation	2.94	±1.29
5. Manage collusion (1): when a patient disagrees with the diagnosis	4.73	±2.06
6. Manage collusion (2): when a patient disagrees with a treatment proposal	4.07	±2.05
7. Manage collusion (3): when a patient has medically inaccurate beliefs	3.62	±1.37
8. Explore a patient’s intense feelings like anger	4.31	±1.55
9. Break bad news to a patient	3.43	±1.58
10. Help a patient deal with the uncertainty of his or her situation	3.25	±1.73
11. Assess symptoms of anxiety and depression	2.81	±1.56
12. Conclude a patient interview with an agreed problem list and a plan for action	3.19	±1.71^a^

^a^Scale from 1 = totally confident to 10 = not at all confident

#### Evaluation of the course

At the end of the training session, the participants completed a feedback form to evaluate the course. We assessed the relevance of the training in terms of clarity (“The content was clear”), newness (“The content was new”), and applicability in medical practice (“The course offered instruments for the implementation of the content”) using a 5-point Likert scale ranging from 1 = strongly agree to 5 = strongly disagree.

Given the limited sample size, we decided to focus on a qualitative evaluation of the course to gain in-depth insight into its perceived value. Two months after the training session, semi-structured phone interviews were conducted with the participants, with the aim of reflecting on the reasons for applying and not applying its contents. The interview guide is presented in Table 4. All interviews were conducted by one researcher (CZ). Data were analyzed using the inductive approach of thematic analysis [50], which allowed us to make sense of the data collected by identifying, analyzing and reporting patterns across the data set. After having familiarized herself with the corpus of interviews, the researcher (CZ) manually generated a first list of codes (semantic content) and organized the data into meaningful groups [51]. A second step consisted of sorting potential themes (repeated patterns) and identifying main themes and sub-themes in relation to the research question. Finally, the researcher checked for internal homogeneity (consistency) and external heterogeneity (distinctiveness) of the themes [52]. To eliminate bias, a second researcher (SR) read and reflected on the transcripts and provided an independent examination of the data. When disagreement over a theme was apparent, the researchers went back to the data and initial coding to compare reasoning and to reach a consensus. Thanks to this method, the researchers could go beyond each participant’s individual experience to build a rich description of the data and construct meaning out of the patterns. As suggested by Lincoln and Guba [44], the researchers kept a journal to reflect on their beliefs, values and assumptions (e.g., in relation to the course design and to the supposed value of the course). An audit trail was also kept to record research steps, including a rationale for decisions about the course design and about data collection, management, and analysis. At the end of the analysis we organized a meeting with three participants in order to summarize and discuss our preliminary findings and to establish the validity of our accounts [44].

**Table 4:** Interview guide for semi-structured phone interviews two months after the training course

General evaluation of the course	After two months, do you think that the content of the course is relevant to the medical practice?
	What did you like most or less?
	Would you like to deepen the topics or do you think that what you have learnt is sufficient?
	Would you advice a colleague to attend this training course?
Strengths and limitations of the training course	What are in your opinion the strengths and limitations of this training course?
	One of your colleagues mentioned in a questionnaire that the content of this course is useful not only in the light of the “information era” but also to better meet the needs of a multicultural or multiethnic society. Do you have any experience in this field? In your daily practice do you see patients with different cultural backgrounds, and does it represent a challenge?
	Do you have any suggestions about how we could improve the training course?
	Some colleagues suggested for instance to gather cases from the participants prior to the training session instead of using standardized cases. What do you think of this idea?
Application of the theory into practice (spontaneously)	In the last two months, do you consider to have applied some of the course content in your medical practice?
	If yes, which ones? Can you give me an example?
	If not, why?
	Was it easy or difficult to apply the content in the practice?
Application of the theory into practice (checklist)	Have you used the checklist during the medical consultations?
	If not, why?
	If yes, how?
	Have you found it difficult or easy to integrate the checklist in your routine practice?
	Have you used it with all patients or only with a specific category of patients?

## Results

### Participants’ assessment of the communication with their patients before the training course

In our baseline questionnaire, we assessed participants’ involvement in prior communication skills training. Between 1995 and 2011, eight participants attended a course, usually a one to two-day course organized by their workplace and focused on communication in medical contexts. However, the participants were unable to provide further details about the content of those courses.

The majority of the participants stated that they were somewhat or very satisfied with their communication with patients. Despite homogeneous satisfaction, half of our participants experienced disagreements with their patients about diagnoses or treatment proposals about once per week or more often.

Participants’ self-efficacy ratings for communication behaviors showed that doctors felt confident in successfully performing a number of communication tasks. On average, they rated 3.40 on a scale from 1 (totally confident) to 10 (not at all confident). This was not surprising considering that our survey population consisted of experts with an average of 27 years of work experience. In particular, our participants felt, on average, very confident in initiating a conversation with their patients about their concerns and in assessing symptoms of anxiety or depression. Despite their experience, the participants recognized the challenge of identifying the reasons behind patients’ viewpoints about their health conditions and about treatments. Dealing with angry patients or with patients who hold medically inaccurate beliefs and communicating bad news are also tasks that our participants felt less confident performing (Table 3). The return rate for the questionnaires was 100 %.

### The value of a course in argumentation theory from the doctors’ perspective

The feedback form collected after the course showed that all doctors considered the content to be relevant, new, and, in principle, applicable to medical practice.

The semi-structured interviews conducted two months after the course aimed to elaborate the reasons for this positive evaluation of the training course. Indeed, in these two months, the participants had the opportunity to reconsider the value of the course in relation to their practice and to implement the checklist. The analysis of the interviews highlighted the strengths and limitations of our approach.

### Strengths of the training course

#### Argumentation to deal with unrealistic expectations and medically inaccurate beliefs

The doctors stated that they often encountered patients who have unrealistic expectations and medically inaccurate beliefs about recovery, treatments, and their role in the management process. The participants considered that putting forward arguments in support of a viewpoint was fundamental because:

*“It is the starting point for engaging in discussion with patients about their expectations for positive outcomes and their views on treatment options”* (D1).

Regarding patients’ medically inaccurate beliefs about the speed of treatment effects, one participant said:

*“Doctors can make plain that they will evaluate the effects of the new therapy in three months because it takes time to alleviate pain when it has lasted for a long time”* (D2).

Similarly, participants affirmed that argumentation could be used to support the need for patients’ engagement in a treatment plan. For example, one participant said:

*“Remember that I can give you weapons, but then you are the player, because medications only help to treat the symptoms”* (D5).

#### Argumentation to anticipate or solve differences of opinion

The participants acknowledged that providing patients with reasons in support of a course of action and checking for their agreement could be a way to anticipate differences of opinion and to foster partnership, especially in situations of uncertainty. According to one participant:

*“When you are not sure about a diagnosis, as is often the case with fibromyalgia, you need to go step by step, share your reasons with your patients, and make sure that they are convinced about your reasons, for instance by asking them ‘Do you agree on doing this medical examination tomorrow to make sure that the problem is what I think it is?’”* (D4).

The participants also observed that the principles of argumentation could guide doctors in their daily practice when a difference of opinion with a patient occurs. For instance, the participants observed that focusing on the reasons for disagreement and avoiding emotional dramatization facilitates its resolution:

*“If you don’t take it personally and you focus on why you disagree, it is easier to discuss issues with patients”* (D7).

Moreover, the participants experienced how beneficial to the doctor–patient relationship it can be to identify and address patients’ disagreement:

*“I prescribed an anti-inflammatory medication to a patient and she suddenly told me not to give her medications that can be bad for her. Then I explained why I thought that this anti-inflammatory drug was good for her”* (D4).

#### Argumentation did not compete with doctors’ actual behavior in practice

The participants reported that reflecting on argumentation as a communication process in doctor–patient interactions was consistent with the procedural know-how they have developed through lessons learned from experience and often through mistakes. This reflection therefore contributed to its legitimization:

*“This course reinforced my idea that it is pointless to speak about complicated diagnoses or diseases. You need to make sure that patients are convinced about what they suffer from and why; you cannot just prescribe, you need to tell them ‘I think this is… because x and y’”* (D4).

Finally, in considering further implementation of the training course, the participants also discussed its value for medical students. They agreed that systematization could allow medical students to move away from purely experiential knowledge by *“providing a plan, a method”* (D1):

*“The course rationalized and built a method out of what we have been doing on a practical, instinctive basis, and this is helpful, especially for young doctors without experience to avoid reproducing our mistakes”* (D9).

### Limitations of the training course

A few participants pointed out several limitations of the course. First, they felt that a one-day course was very intensive and did not give them enough time to digest the information and that more educational meetings would support better integration into their practice. For example, one participant said:

*“I’m not saying that I forgot about the course, but when you are busy, you forget; without continuity, the course risks being a nice thing and nothing more”* (D10).

Second, the participants made suggestions about conducting the exercise section of the course through video-recorded role play, as looking at videos can incentivize the confrontation among colleagues.

Third, one participant disagreed with the concept of argumentation as a whole because, in his view, the fact that the doctor had to justify his point of view weakens his authority:

*“I don’t want to assume that I’m the doctor and that by definition I’m right, but we cannot even assume that I need to make a huge effort to make the patient understand that he or she is wrong because until proven otherwise, I am the doctor. Of course I can be wrong, but I didn’t study 15 years for nothing!”* (D10).

Similarly, another participant stated that the roles of doctor and patient should always be clearly defined: *“I don’t have to adapt to you; I’m the doctor, you’re the patient”* (D1). One of the participants explained that this paternalistic approach might be an expression of the Italian context where in many regions, doctors visit few foreign patients:

*“In Italy, argumentation might not be relevant to every doctor yet, because the need to explain the reasons for a medical recommendation is stronger in a multicultural context where people have different expectations from doctors”* (D11).

## Discussion

In this paper, we attempted to contribute to the research in the fields of medical education and argumentation theory. More specifically, the aim was to build a bridge between these two streams of research by showing how medical education can benefit from specific training courses in argumentation. Although the participants were satisfied with their communication with patients and had a high level of self-efficacy in communicating with them before the training course, they nevertheless found that the contents of the course were relevant for and applicable to medical practice. On the one hand, the course acknowledged the expertise that doctors have acquired based on their experience. On the other hand, it taught them techniques to optimize this expertise and addressed some of the challenges of modern doctor–patient interactions. More specifically, the participants stated that argumentation is useful during consultations to deal with unrealistic expectations and medically inaccurate beliefs and to solve differences of opinion.

In our view, these findings are relevant and important for five main reasons. First, they show that the use of argumentation in medical encounters can build bridges between doctors and patients. It fosters their collaboration by favoring the expression and testing of their respective points of view. This role of argumentation is indirectly supported by the evidence that doctors, while avoiding artificial neutrality, should offer supported recommendations to promote informed autonomy [45]. In addition, the use of argumentation in doctor–patient communication is a way to take into consideration the patient perspective that the literature on person-centered healthcare recognizes as a key element for the successful development and management of therapeutic plans.

Second, training in argumentation seems to be appropriate to instruct on how to deal with conflict in doctor–patient communication. As stated by Katz [53], conflict is inevitable in every relationship. Conflict includes a variety of situations, from overt disagreement to submerged tensions [54, 55]. Wolf’s [56] analysis highlighted that in medical consultations, doctors and patients have applied different strategies to avoid or minimize conflict. For instance, doctors minimize conflict by presenting patients only one option [6, 57], and patients do so by withholding their differences of opinion [58–60]. Training in argumentation can help normalize disagreements between doctors and patients and allows patients’ perspectives to enter the medical consultation without fear of a reduced quality of care [53]. According to Rosser and colleagues [61], training practitioners to identify and address patients’ attitudes and beliefs about medications would improve adherence with medical recommendations and facilitate doctor–patient interaction. At the same time, by supporting their diagnosis with reasons, doctors avoid presenting themselves as undisputed authorities [62].

Third, building on the previous two points, the value of training doctors in argumentation seems to be in line with current findings from other fields, particularly science education [63]. As in the training of scientists, argumentation has the potential to inform doctors on how to conduct an “accountable talk” [63–65] with defined norms of discussion. These norms, on the one hand, can build structure in daily experiences of communication and, on the other hand, can promote doctors’ self-reflection on the specific communication moves that facilitate or hinder mutuality and collaboration with patients in decision–making.

Fourth, training in argumentation can help doctors in recognizing the value of argumentation in education [66]. By mastering argumentative discussion skills, they can argue various aspects including what counts as a good or bad reason for a claim, when claims are relevant or irrelevant to a standpoint, and what conclusion to follow based on different kinds of evidence. Overall, they acquire normative standards to be able to conduct the discussion in a reasonable way. As Chin [67] points out, the role of the physician should not be relegated to that of an information–provider, as this would play down the intrinsic inequality of knowledge and skills between doctors and patients. Considering the doctor a simple information–provider dismantles the whole nature of the doctor–patient relationship that rests on the assumption that the patient seeks assistance from the doctor who is able to provide it. An open dialogue in which doctors frankly admit their standpoints is ultimately a better protector of patients’ right to autonomous choice than artificial neutrality [45]. By constructing arguments doctors do not patronize the interaction (as they would do if they imposed their views without supporting them with reasons), but rather they expose their standpoints in interactions with patients to be evaluated and pondered by patients.

Fifth, our findings indicate that there might be countries, such as Italy, where an argumentative interaction might still be perceived as a devaluation of doctors’ expertise. Our findings are consistent with recent studies conducted in Italy in the field of patient-centered care and shared decision–making that show that Italian doctors tend to explore patients’ illness experiences only when patients display their willingness [68]. Another Italian study conducted in the field of nephrology showed that only 29 % of doctors believed that the decision regarding dialysis treatment in end-of-life patients should be shared with patients and their families [69]. Studies conducted in Italy show that, in certain fields, patients still prefer a passive role in the consultation [70–72]; this could explain why the paternalistic approach is still perceived as the most appropriate model.

Our study has several limitations. It is a pilot study conducted in only one setting with a small, convenient and self-selected sample of participants; for this reason, generalizations of findings are not possible. We cannot exclude the fact that only those doctors who were favorable to communication as a means to improve care agreed to participate in the training course, thus enhancing the chances of a positive evaluation. In addition, the participants were aware that the teachers were involved in the evaluation of the training course, and in reporting on their behaviors; therefore, they might have been prone to give socially desirable answers. However, the fact that they were able to illustrate, with examples from their practice, the strengths of the training course supports the frankness of their answers. For future research, we suggest conducting the course in more than one setting and structuring it in multiple sessions (with one to two months in between sessions)—with an emphasis on the presentation of case studies in which argumentative discussion skills have been successfully or unsuccessfully implemented—where doctors can share their experiences and identify solutions. The suggested improvement in the course organization will allow another limitation of our study to be overcome, namely the difficulty of integrating new professional habits and establishing new professional routines. It has been shown that changing work habits involves a paradigm change, namely giving up habits learned during educational and professional socialization in favor of new behaviors [73]. Structuring the course over more than one session supports the implementation of new behaviors [74], therefore allowing an evaluation of the transfer of skills from theory to practice.

## Conclusions

This paper enriches the research in the field of medical education by showing how and why doctors and health professionals in general can benefit from a specific training course in argumentation. It directs attention to the value of argumentation in managing the differences of opinion that arise in the confrontation between doctors’ and patients’ perspectives. The study of argumentation can provide doctors with techniques to address patients’ viewpoints on health conditions and treatments. Moreover, given that integrating new habits into practice is challenging [73], this study shows that argumentation as a communication process is in line with doctors’ actual behavior in practice. Ultimately, this paper supports the value of training doctors to strengthen communication skills that go back to a tradition of classical rhetoric, which focused on empowering individuals. Until a few decades ago, the dominant paternalistic model of doctor–patient communication minimized the application of argumentation, but in the new era of shared decision–making and patient autonomy, argumentation has become a key communication process. By using argumentation, doctors and patients can exchange their viewpoints and reach an agreement about what to do. Moreover, through the rational exchange that is typical of argumentation, medically inaccurate beliefs can be discussed and can better serve as a basis for improved patient decisions.
